# Influence of AI ethics awareness, attitude, anxiety, and self-efficacy on nursing students’ behavioral intentions

**DOI:** 10.1186/s12912-022-01048-0

**Published:** 2022-09-30

**Authors:** Yeunhee Kwak, Jung-Won Ahn, Yon Hee Seo

**Affiliations:** 1 Red Cross College of Nursing, Chung-Ang University, 84, Heukseok-ro, Dongjak-gu, 06974 Seoul, Korea; 2 Department of Nursing, Gangneung-Wonju National University, 150, Namwon-ro, Heungeop-myeon, 26403 Wonju-si, Gangwon-do Korea; 3 Department of Nursing, Yeoju Institute of Technology, 338, Sejong-ro, 12652 Yeoju-si, Gyeonggi-do Korea

**Keywords:** AI ethics awareness, Attitude toward AI, Anxiety, Self-efficacy, Behavioral intention

## Abstract

**Background:**

Artificial intelligence (AI) technology has recently seen rapid advancement, with an expanding role and scope in nursing education and healthcare. This study identifies the influence of AI ethics awareness, attitude toward AI, anxiety, and self-efficacy on nursing students’ behavioral intentions to use AI-based healthcare technology.

**Methods:**

The participants included 189 nursing students in Gyeonggi-do, with data collected from November to December 2021 using self-reported questionnaires. We analyzed the data using the SPSS/WIN 26.0 program, including a t-test, Pearson’s correlation coefficient, and hierarchical multiple linear regression.

**Results:**

The results revealed that AI ethical awareness (t = − 4.32, *p* < .001), positive attitude toward AI (t = − 2.60, *p* = .010), and self-efficacy (t = − 2.65, *p* = .009) scores of the third and fourth-year nursing students were higher, while their anxiety scores were lower (t = 2.30, *p* = .022) compared to the scores of the first and second-year nursing students. The factors influencing behavioral intention included a positive attitude toward AI (β = 0.58) and self-efficacy (β = 0.22). The adjusted R^2^ was 0.42.

**Conclusion:**

It is necessary to inculcate a positive attitude toward AI and self-efficacy by providing educational programs on AI-based technology in healthcare settings.

## Background

Artificial intelligence (AI) is a crucial technology that will play a leading role in the fourth industrial revolution. To date, many experts have discussed the role and scope of AI in dramatically increasing diagnostic efficiency in medical practice, making accurate decisions, and addressing the issue of medical staffing shortages [1, 2]. Integrating AI technology in healthcare products and services will enable healthcare professionals to use AI for patient monitoring, diagnosis, and treatment and help integrate various environmental, genomic, health, and sociodemographic data. In addition, integrating data and enhancing information utilization with AI-based technology will contribute to achieving a higher standard of nursing care, strengthening nurses’ competencies, and providing evidence-based individual quality of nursing care to meet patient goals and priorities [3].

The use of AI in nursing has already taken place in the analysis of electronic nursing records, clinical decision support through analysis of pressure sores and safety risks, nursing robots, and scheduling [2, 3]. However, despite positive performance and utilization expectations from introducing and expanding AI technology in the healthcare setting, there are growing concerns. These concerns relate to various unpredictable problems, discrimination and ethical issues caused by malfunctions and incomplete technology of AI medical devices, distortion and bias of information due to lack of accumulated data or learning errors in AI, and invasion of privacy is also growing [4–6]. AI ethics are the behaviors, lifestyles, principles, and technologies that transcend national boundaries, upheld among individuals, companies, societies, and between robots [7]. Increased awareness of AI ethics led to national and international AI ethical standards [8, 9]. Evaluating nursing students’ AI ethics subsequently enables assessing educational needs and introducing relevant content into the nursing education curriculum.

Meanwhile, people’s attitudes toward AI play an important role in accepting AI. For instance, Wagner and Sherwood [10] reported that a learner’s positive attitude toward information technology (IT) reduced their anxiety about IT, promoted IT use or interactions, and improved confidence in problem-solving through IT. Additionally, attitudes toward IT affected emotions, behaviors, and ideas about IT, emphasizing the learners’ importance of a positive attitude toward IT. Furthermore, studies have shown that more experience using IT, such as AI, can lead to less fear [11] and anxiety [12] for a better understanding of AI. Subsequently, students familiar with AI tended to show more optimistic and passionate attitudes toward AI-related work than their counterparts [13]. Therefore, to resolve the ethical issues and conflicts associated with AI, proper understanding of AI, and awareness and education regarding ethical decision-making standards, must come first. Thus, nurses can escape their anxiety and negative attitudes toward AI and recognize AI as a co-existing healthcare technician to minimize confusion and build trust among patients and caregivers.

Nurses and nursing students are potential users of AI-based technology and are in a pivotal position to shape and lead the use of AI within the nursing field [14]. However, a recent study reported that more than 70% of nurses and nursing students did not understand AI in clinical practice. This thinking is despite believing that AI will revolutionize nursing and healthcare by improving health promotion and disease prevention, support for establishing personalized treatment plans, work automation, and teamwork between professionals [15]. In other words, while nurses demonstrated a high degree of awareness regarding the usefulness and efficiency of AI technology, no studies have identified the self-efficacy, attitude, and intention to use AI in providing nursing care. Moreover, with the rapid evolution of AI-based technology and demand for AI education in nursing, we require developing and advancing nurses’ job competencies and roles to enable them to learn relevant knowledge and increase their understanding of extensive data and utilization [16]. Consequently, there is a need to understand nursing students’ attitudes, self-efficacy, and intentions to use AI to educate prospective nurses to lead and adapt to these changes in technology in nursing. Therefore, this study assessed nursing students’ ethical awareness, attitude, anxiety, and self-efficacy toward AI and identified the factors influencing behavioral intention to use AI-based healthcare technologies.

## Method

### Study Design

We conducted a cross-sectional study to identify factors affecting nursing students’ behavioral intentions to use AI-based healthcare technology.

### Study participants

The participants were 189 nursing students in Gyeonggi-do, Korea. We obtained permission for participant recruitment from the dean of a nursing department at a local university in Gyeonggi-do. The target participants were first to fourth-year nursing students. We posted a recruitment note on the nursing notice board for ten days. Then a research assistant visited classrooms during break time, verbally explained the purpose of the study, and distributed questionnaires to nursing students who voluntarily agreed to participate. We recruited those who understood the purpose of this study and provided consent for participation. We collected data from February 16−23, 2022, and determined the sample size using G*Power software (version 3.1.7, Heinrich-Heine-University, Germany). For multiple regression analysis, we used behavioral intention as a dependent variable. Based on the effect size of 0.15, significance level (⍺) of 0.05, statistical power (1-β) of 0.95, and 11 arbitrary predictors (six demographic characteristics, AI ethics awareness, two attitudes, anxiety, and self-efficacy), we calculated the minimum sample size as 178. We distributed the questionnaires to 195 students. We had 189 questionnaires in the final analysis, indicating a dropout rate of 3.1%; we excluded one questionnaire with insincere responses, one with an abnormal Z-score, and four with outliers in the regression analysis.

## Measurements

### AI Ethics Awareness

We measured AI ethics awareness using the Test for Artificial Intelligence Ethics Awareness (TAIEA), a tool developed by Kim and Shin [6]. It consists of 24 items in eight categories (responsibility, stability and reliability, no discrimination, transparency and explainability, human-centered service, employment, allowance and restriction, and robot rights). We used a five-point Likert scale (1 point for “*strongly disagree*” to 5 points for “*strongly agree*”) to rate each item. The higher the score, the higher the level of ethical awareness. Cronbach’s α of the tool was 0.81 at the time of development [6] and 0.67 in this study.

#### General Attitudes toward Artificial Intelligence Scale (GAAIS)

We used the GAAIS to measure attitude toward AI. The measurement scale comprised 20 items, including 12 positive and eight negative attitudes toward AI [17]. After obtaining approval from the original developer of the tool, we translated and back-translated it for use. Participants rated each item on a five-point Likert scale (1 point for “*strongly disagree*” to 5 points for “*strongly agree*”). A higher score in a positive category means that participants have a more positive attitude. We did not reverse-score the items on negative attitudes; therefore, higher scores in a negative category meant that participants had more negative attitudes toward AI. The Cronbach’s α of the positive and negative attitudes were 0.88 and 0.83, respectively, at the time of development [17], and 0.85 and 0.76, respectively, in this study.

#### Anxiety

We used questions from the Technology Acceptance Model (TAM) developed by Venkatesh et al. [18] to measure anxiety. In addition, we changed the term “technology” and/or “system” to “AI-based technology within the healthcare setting.” The tool comprised four items on anxiety regarding the use of AI technology, and participants rated each item on a five-point Likert scale (1 point for “*strongly disagree*” to 5 points for “*strongly agree*”). Cronbach’s α of the tool was ≥ 0.70 in previous studies [18, 19] and 0.86 in this study.

#### Self-efficacy

We used questions from the TAM by Venkatesh et al. [18] to measure self-efficacy, consisting of four items. Participants rated each item on a five-point Likert scale (1 point for “*strongly disagree*” to 5 points for “*strongly agree*”), with higher scores indicating higher self-efficacy in using AI-based technology. Cronbach’s α of the tool was ≥ 0.80 in previous studies [18, 19] and 0.75 in this study.

#### Behavioral intention

We applied questions from the TAM by Venkatesh et al. [18] to measure behavioral intention to use AI-based technology. Participants rated three items on a five-point Likert scale (1 point for “*strongly disagree*” to 5 points for “*strongly agree*”), with higher scores indicating higher behavioral intention to use AI-based technology. Cronbach’s α of the tool was ≥ 0.80 in a previous study [19] and 0.66 in this study.

### Ethical considerations

We conducted this study after receiving the institutional review board’s approval (1041078-202112-HR-342-01). We informed all voluntary participants about the purpose of the research and ensured that all responses were anonymous. Data collected using questionnaires were coded and entered into a database without personally identifiable information. We compensated all participants for their participation.

### Data Analysis

We used the SPSS WIN 26.0 Program (IBM Corp., Armonk, NY, USA) for data analysis. First, we analyzed participants’ general characteristics using frequency analysis and descriptive statistics. Next, we analyzed the measured variable scores according to general characteristics using t-tests and the correlation between variables using Pearson’s correlation coefficient. Finally, we confirmed the factors affecting nursing students’ behavioral intentions to use AI-based healthcare technology using hierarchical multiple linear regression analysis.

## Results

### General characteristics of the participants

The participants included 162 females (85.7%, M_age_ = 23.89); the majority were second-year students (n = 123; 65.1%). The average Internet usage time, excluding the time used for learning purposes, was 4.27 h. The main reasons for using the Internet were messenger/social networking services, watching movies/TV/videos, searching for information, and playing games, in that order. The group included 36 students (19.0%) who had participated in AI-related education (Table 1).

**Table 1:** General Characteristics of Participants (N = 189)

Characteristics	Categories	n (%) or M ± SD
Gender	Men	27(14.3)
	Women	162(85.7)
Age (years)		23.89 ± 6.94
Education year	Year 1	2(1.1)
	Year 2	123(65.1)
	Year 3	48(25.4)
	Year 4	16(8.5)
Internet use time per day (hours)		4.27 ± 2.09
Purpose of using Internet^*^ (n = 447)	Using messenger/social networking services	150(33.5)
	Playing games	40(9.0)
	Obtaining information	113(25.3)
	Watching movies/TV/videos	144(32.2)
AI education experience	Yes	36(19.0)
	No	157(81.0)

^*^Multiple responses

### Differences in measured variables by demographic characteristics

Analysis of the measured variables according to demographic characteristics showed no statistically significant differences in AI ethics awareness, positive and negative attitudes toward AI, anxiety, self-efficacy, and behavioral intention scores according to gender, age, Internet use time, and AI education experience. However, the third and fourth-year students showed significantly higher AI ethics awareness, positive attitudes toward AI, self-efficacy scores, and significantly lower anxiety scores than first and second-year students. In addition, the behavioral intention score of the third and fourth-year nursing students was higher than in other years, and the difference in scores was close to the significance level (*p* = .053) (Table 2).

**Table 2:** Differences in the Measured Variable By Demographic Characteristics (N = 189)

Characteristics	Categories	n (%)	AI ethics awareness	t (*p*)	Positive attitude toward AI	t (*p*)	Negative attitude toward AI	t (*p*)	Anxiety	t (*p*)	Self- efficacy	t (*p*)	Behavioral intention	t (*p*)
			M ± SD		M ± SD		M ± SD		M ± SD		M ± SD		M ± SD	
Gender	Men	27(14.3)	2.69 ± 0.55	−0.375 (0.708)	3.71 ± 0.59	0.452 (0.654)	2.89 ± 0.59	−0.655 (0.513)	3.20 ± 1.00	−0.511 (0.610)	3.57 ± 0.67	0.377 (0.709)	3.83 ± 0.69	0.620 (0.536)
	Women	162(85.7)	2.73 ± 0.47		3.65 ± 0.45		2.96 ± 0.54		3.29 ± 0.78		3.52 ± 0.53		3.76 ± 0.54	
Age	≤ 30	166(87.8)	2.73 ± 0.48	0.458 (0.648)	3.66 ± 0.48	0.248 (0.804)	2.95 ± 0.55	−0.538 (0.591)	3.31 ± 0.82	1.481 (0.140)	3.52 ± 0.57	−0.426 (0.671)	3.76 ± 0.57	−0.290 (0.772)
	31-	23(12.2)	2.68 ± 0.46		3.64 ± 0.42		3.01 ± 0.54		3.04 ± 0.77		3.58 ± 0.40		3.80 ± 0.48	
Education year	Year 1&2	125(66.1)	2.62 ± 0.49	−4.315 (< 0.001)	3.60 ± 0.46	−2.595 (0.010)	3.00 ± 0.54	1.473 (0.142)	3.37 ± 0.81	2.302 (0.022)	3.46 ± 0.58	−2.652 (0.009)	3.71 ± 0.56	−1.945 (0.053)
	Year 3&4	66(33.9)	2.91 ± 0.40		3.78 ± 0.47		2.87 ± 0.56		3.09 ± 0.79		3.68 ± 0.56		3.88 ± 0.54	
Internet use time per day (hours)	≤ 4	109(57.7)	2.70 ± 0.47	−0.572 (0.568)	3.68 ± 0.46	0.785 (0.433)	2.94 ± 0.51	−0.504 (0.615)	3.24 ± 0.82	−0.684 (0.495)	3.55 ± 0.51	0.586 (0.559)	3.74 ± 0.55	−0.640 (0.523)
	4<	80(42.3)	2.74 ± 0.50		3.63 ± 0.48		2.98 ± 0.60		3.33 ± 0.81		3.50 ± 0.60		3.80 ± 0.57	
AI education experience	Yes	36(19.0)	2.70 ± 0.50	−0.219 (0.827)	3.71 ± 0.44	0.644 (0.520)	3.03 ± 0.50	0.908 (0.365)	3.16 ± 0.84	−0.969 (0.334)	3.53 ± 0.65	−0.032 (0.974)	3.77 ± 0.55	0.037 (0.971)
	No	153(81.0)	2.72 ± 0.48		3.65 ± 0.48		2.94 ± 0.56		3.31 ± 0.81		3.53 ± 0.52		3.76 ± 0.56	

### Correlations between AI Ethics Awareness, attitude toward AI, anxiety, Self-Efficacy, and behavioral intention

Table 3 describes the correlations between AI ethics awareness, attitude toward AI, anxiety, self-efficacy, and behavioral intention. There were statistically significant correlations among all measured variables. Positive attitude toward AI (*r* = − .62) and self-efficacy (*r* = .51) showed the highest correlation with behavioral intention. Self-efficacy positively correlated with AI ethics awareness (*r* = − .25) and negatively correlated with negative attitudes toward AI (*r* = − .31) and anxiety (*r* = − .34). Anxiety had a positive correlation with a negative attitude toward AI (*r* = .44). In contrast, AI ethics awareness had a positive correlation with a positive attitude toward AI (*r* = .31) (Table 3).

**Table 3:** Correlations between Measured Variables (N = 189)

Variables	M ± SD	1	2	3	4	5	6
		*r* (*p*)					
1	3.27 ± 0.24	1					
2	3.66 ± 0.47	0.52 (< 0.001)	1				
3	2.95 ± 0.55	−0.15 (< 0.001)	−0.23 (0.001)	1			
4	3.28 ± 0.81	−0.13 (0.020)	−0.19 (0.010)	0.44 (< 0.001)	1		
5	3.53 ± 0.55	0.35 (0.001)	0.54 (< 0.001)	−0.31 (< 0.001)	−0.34 (< 0.001)	1	
6	3.77 ± 0.56	0.31 (0.009)	0.62 (< 0.001)	−0.24 (0.001)	−0.27 (< 0.001)	0.51 (< 0.001)	1

*Row and column headings*: 1 ***=*** AI ethics awareness, 2 = Positive attitude toward AI, 3 = Negative attitude toward AI, 4 = Anxiety, 5 = Self-efficacy, 6 = Behavioral intention

### Factors Associated with the behavioral intention to use AI-based Healthcare Technologies

We performed linear regression analysis to analyze the effects of AI ethics awareness, positive and negative attitudes toward AI, anxiety, and self-efficacy—which showed significant differences in ANOVA and correlation analysis—on behavioral intention. The regression model was statistically significant (F = 28.38, *p* < .001) and had an explanatory power of 42.1%. The results showed no problem with multicollinearity with a variance inflation factor (VIF) of 1.30–1.71. Moreover, the Durbin-Watson test showed *d* = 1.91, indicating no autocorrelation of residuals. The regression analysis showed that a positive attitude toward AI (β = 0.58) and self-efficacy (β = 0.22) were the two variables that had a significant influence on nursing students’ behavioral intentions to use AI-based healthcare technology. The results showed R^2^ and adjusted R^2^ of 0.44 and 0.42, respectively (Table 4).

**Table 4:** Factors Associated with Behavioral Intention (N = 189)

Variable	Behavioral Intention				
	B	SE	β	t	*p*
Constant	1.41	0.52		2.73	0.007
AI ethics awareness	−0.08	0.15	−0.04	−0.54	0.590
Positive attitude toward AI	0.58	0.09	0.49	6.77	< 0.001
Negative attitude toward AI	−0.02	0.06	−0.02	−0.32	0.747
Anxiety	−0.07	0.04	−0.11	−1.65	0.102
Self-efficacy	0.22	0.07	0.22	3.15	0.002
R^2^	0.44				
Adjusted R^2^	0.421				
F-value (*p*)	28.38 (< 0.001)				

## Discussion

This study evaluated the level of ethical awareness, attitude toward AI, anxiety, and self-efficacy and identified the factors influencing nursing students’ intentions to use AI-based health technology. As a result of analyzing the measurement variables according to the general characteristics, the third and fourth-year students showed significantly higher AI ethics awareness, positive attitudes toward AI, and self-efficacy, and significantly lower anxiety scores than the first and second-year students. In addition, the difference in behavior intention scores was also close to the significance level. The nursing education curriculum in Korea specifies that third and fourth-year students participate in clinical practice training. Clinical practice requires a basic knowledge of technologies related to nursing skills and medical equipment. Therefore, students encounter AI technology applications in health settings during such classes based on active learning tasks that familiarize them with various technologies and advance their nursing skills and theoretical knowledge. This exposure to AI technologies and build-up of experience creates a positive attitude toward the perceived usefulness of technology. It can also improve the students’ intentions to use AI technology [19]. A previous study reported that fourth-year nursing students showed the most positive attitudes toward technology, as they had already had more opportunities to participate in technology education [20]. This result could be from expectations and familiarity with healthcare technology among third and fourth-year nursing students who frequently have direct and indirect experiences with AI health technologies (AIHTs) in clinical practice, leading to increased positive attitudes toward AI. As a result, this attitude positively affects AI ethics awareness, self-efficacy, and behavioral intention, ultimately reducing anxiety.

This study showed a positive correlation between AI ethics awareness and positive attitudes toward AI, self-efficacy, and behavioral intention. On the other hand, AI ethics awareness negatively correlated with negative attitudes toward AI and anxiety. According to the EU’s “Potential Benefits of Artificial Intelligence and Ethical Considerations” brief report, using AI requires effort through appropriate regulations, ethical considerations, education, and research to achieve the purpose of good intentions [21, 22]. Regarding AI ethics awareness, firmer ideal ethical beliefs raise greater awareness about the applicability of AI, ethical dignity of AI, and fairness of AI ethics; stronger relative ethical beliefs tend to lower the attention to the adverse effects of AI [23]. In the coming intelligent information society led by AI, one must include AI ethics in the nursing curriculum to allow more individuals to benefit from technology and minimize dysfunction and adverse effects associated with AI [24]. Anxiety factors have a negative impact on the intention to use AI. Providing nursing students information on the usefulness and benefits of AI technology can improve their choice to use AI and their acceptance of AI-based technology [19].

Approximately 81% of the participants in this study responded that they had no experience in AI education; therefore, it would have been difficult to induce changes in the subject’s ethical awareness, attitudes, and self-efficacy toward AI. Only a few studies on AI ethics included a wide range of healthcare workers. Studies that identified and compared the ethical values among healthcare workers and ethical standards for new AI technologies are even more scarce. Accordingly, it is necessary to provide basic data related to AI ethics through replication studies. In previous studies on medical students in the United States and the United Kingdom, few students had received an AI education. Most students responded that the current education was not sufficiently helpful in preparing for new technologies in healthcare, despite their awareness of the importance of AI and related education [25, 26]. With AI becoming a vital part of nursing and healthcare, researchers recognize the need for AI-related knowledge, skills, and competencies. However, previous studies have found that students’ knowledge and technical competencies fell short of the levels needed in clinical practice [3, 15].

These results confirm the insufficiencies in nursing education regarding students’ abilities to use AI-based healthcare technologies, indicating the need for developing nursing education programs in educational institutions and clinical practices that would allow students to participate in practical training safely and effectively [27]. These findings also suggest the need for education in data literacy, technical literacy, systems thinking, AI algorithms, and AI’s ethical meaning to improve nursing competency [21, 28]. Educating nursing students in AI is essential, including the ethical use of AI and technology and protecting patients from potential threats [5, 8]. As AI utilization expands to improve human convenience, developing and enhancing awareness of the world’s common AI ethical principles and providing related ethics education is necessary. Furthermore, AI can directly or indirectly affect patients’ health management, quality of life, and prognosis in healthcare settings. Thus, a systematic education program to promote understanding, use, and ethical education in AI will strengthen the nursing competency that can actively adapt to patient protection and rapid changes in the healthcare environment.

This research showed that positive attitudes toward AI and self-efficacy influenced nursing students’ behavioral intentions to use AI-based technologies. Attitudes toward IT influence emotions, behaviors, and ideas about IT; thus, learners need to have a positive attitude toward IT [10]. Increased awareness of AI technology use leads to active behavioral intention and attitude, while attitude influences experience, self-efficacy, motivation, and beliefs [12, 13, 19]. We found that attitudes affected learners’ academic achievements and various factors such as efficacy, motivations, and beliefs [29]. Learners with a positive attitude toward IT may be more motivated to learn using IT and can develop various IT-related ideas. As such, as the use of IT increases, anxiety about IT will decrease, forming a positive attitude [20]. Regarding technology acceptance, self-efficacy can change behavioral intentions by raising awareness about technology’s ease of use and sufficient knowledge [30]. From a technology acceptance perspective, self-efficacy can enhance understanding regarding ease of use, which can positively influence behavioral intention to use [30, 31]. Positive awareness of AIHTs among nursing managers influenced positive attitudes, while nursing educators played a crucial role in preparing nurses and nursing students for AIHTs [27, 32]. Therefore, it is necessary to enhance self-efficacy and positive attitudes by providing educational programs on AIHTs.

Further, the study findings showed that AI ethics awareness did not significantly influence behavioral intention. Regarding the TAIEA tool used in this study, the researchers initially only reported expert validity and reliability. Accordingly, we deleted many items for construct validation in this study. Nevertheless, the internal reliability of the tool was only 0.69, indicating a need for the tool’s further revision and verification. Therefore, we need follow-up studies on developing research tools and AI ethics awareness.

Nursing students’ positive attitudes toward AI and improvements in self-efficacy can increase their behavioral intentions toward using AI-based healthcare technology applications. Providing nursing students with education on AI technology’s performance, usefulness, and social influence in health settings will thus lead to a positive attitude. It will also provide an opportunity to directly and indirectly experience AI-based healthcare technology and intervention in clinical settings, increase nurses’ self-efficacy, and improve their behavioral intentions.

This study had some limitations. First, we collected data from nursing students at a single nursing college, and most participants did not have previous experience in AI education. Therefore, there are limitations in generalizing this study’s findings. Second, when researchers initially developed the TAIEA tool, they did not perform factor analysis. Thus, we excluded items with low factor loading after factor analysis for item analysis and construct validation from the analysis. Accordingly, to improve the accuracy and efficiency of the measurement tool, we need follow-up studies after developing a tool for measuring AI ethics awareness that reflects the characteristics of nursing and healthcare fields. Finally, to develop competencies among nursing students to use AI-based technology and meet the demands of the times, it is necessary to create an innovative undergraduate curriculum with opportunities to practice digital healthcare technologies, including AI and AI ethics and algorithms.

## Conclusion

In this study, only 19.1% of the participants had experience with AI-related education. However, students with practical clinical training showed significantly higher AI ethics, positive attitudes toward AI, self-efficacy, and behavioral intention scores while having significantly lower anxiety scores than first and second-year students. Moreover, we identified positive attitudes toward AI and self-efficacy as influencing factors on behavioral intention. Such findings indicate the need for enhancing positive attitudes toward AI and self-efficacy through educational programs on AI technology in healthcare settings. Moreover, there is also an urgent need for nursing curriculum reform in educational institutions and clinical practice that would allow nursing students to participate in practical training safely and effectively in this age of AI. Therefore, it is necessary to increase the educational experience related to AI. In particular, it is imperative to develop and apply various educational programs based on ethical awareness, relevant information, and the usage of AI technology in healthcare settings before nurses enter clinical practice. In addition, introducing AI content in the nursing curriculum will improve nursing students’ positive attitudes and self-efficacy toward AI technology within the clinical field.

## Data Availability

The datasets used and/or analyzed during this study are available from the corresponding author upon reasonable request.

## Abbreviations

AI: artificial intelligence
AIHT: artificial intelligence health technology
GAAIS: General Attitudes towards Artificial Intelligence Scale
IT: information technology
TAIEA: Test for Artificial Intelligence Ethics Awareness
TAM: Technology Acceptance Model
VIF: variance inflation factor
